# RAGE-TXNIP axis drives inflammation in Alzheimer’s by targeting Aβ to mitochondria in microglia

**DOI:** 10.1038/s41419-022-04758-0

**Published:** 2022-04-04

**Authors:** Oualid Sbai, Mehdi Djelloul, Antonia Auletta, Alessandro Ieraci, Carlo Vascotto, L. Perrone

**Affiliations:** 1 Caminnov sas, Montpellier, France; 2 University Aix-Marseille, Marseille, France; 3 Department of Advanced Medical and Surgical Sciences, 2nd Division of Neurology, Center for Rare Diseases and InterUniversity Center for Research in Neurosciences, University of Campania Luigi Vanvitelli, Naples, Italy; 4 Department of Pharmaceutical Sciences, University of Milan, Milan, Italy; 5 Department of Medicine, University of Udine, Udine, Italy; 6 DKFZ, Department of Functional and Structural Genomics, Heidelberg, Germany; 7 University of Poitiers, Poitiers, France

**Keywords:** Cellular neuroscience, Inflammasome

## Abstract

Alzheimer’s disease (AD) is the most common form of dementia characterized by progressive memory loss and cognitive decline. Although neuroinflammation and oxidative stress are well-recognized features of AD, their correlations with the early molecular events characterizing the pathology are not yet well clarified. Here, we characterize the role of RAGE–TXNIP axis in neuroinflammation in relation to amyloid-beta (Aβ) burden in both in vivo and in vitro models. In the hippocampus of 5xFAD mice microglial activation, cytokine secretion, and glial fibrillary acidic protein-enhanced expression are paralleled with increased TXNIP expression. TXNIP silencing or its pharmacological inhibition prevents neuroinflammation in those mice. TXNIP is also associated with RAGE and Aβ. In particular, RAGE–TXNIP axis is required for targeting Aβ in mitochondria, leading to mitochondrial dysfunction and oxidative stress. Silencing of TXNIP or inhibition of RAGE activation reduces Aβ transport from the cellular surface to mitochondria, restores mitochondrial functionality, and mitigates Aβ toxicity. Furthermore, Aβ shuttling into mitochondria promotes Drp1 activation and exacerbates mitochondrial dysfunction, which induces NLRP3 inflammasome activation, leading to secretion of IL-1β and activation of the pyroptosis-associated protein Gasdermin D (GSDMD). Downregulation of RAGE–TXNIP axis inhibits Aβ-induced mitochondria dysfunction, inflammation, and induction of GSDMD. Herein we unveil a new pathway driven by TXNIP that links the mitochondrial transport of Aβ to the activation of Drp1 and the NLRP3 inflammasome, promoting the secretion of IL-1β and the pyroptosis pathway associated with GSDMD cleavage. Altogether these data shed new light on a novel mechanism of action of RAGE–TXNIP axis in microglia, which is intertwined with Aβ and ultimately causes mitochondria dysfunction and NLRP3 inflammasome cascade activation, suggesting TXNIP as a druggable target to be better deepened for AD.

## Introduction

Alzheimer’s disease (AD) is the most common neurodegenerative disorder and the prevalent cause of dementia in the elderly [1]. The brain of AD patients is characterized by extracellular plaques of amyloid-beta peptide (Aβ) and neurofibrillary tangles of hyperphosphorylated tau [2]. Recently, more attention has been addressed to the causative role of neuroinflammation and oxidative stress in AD. Indeed, Aβ is both cause and consequence of neuroinflammation and oxidative stress [3]. Introducing new awareness of the upstream pathways involved in this vicious circle could highly contribute to AD research.

Neuroinflammation mediated by microglia plays a key role in AD pathogenesis [4]. Microglia drives innate immunity surveillance in the central nervous system. When chronically activated, microglia become dysfunctional [5] and promotes neurodegeneration [6]. Misfolded proteins, such as Aβ, activate the innate immune response, leading to the release of inflammatory cytokines, which participate in AD progression [7]. A critical component of the innate immune system is the Nod-like receptor protein 3 (NLRP3). NLRP3 has a central role in AD pathophysiology [8] by activating pro-caspase-1 in response to cell damage [9]. Such activation promotes the secretion of proinflammatory cytokines IL-1β/IL-18 [10] and also the cleavage of Gasdermin D (GSDMD), a key protein of the pyroptosis cascade—a type of cell death triggered by proinflammatory signals [11]. Oxidative stress activates the NLRP3 inflammasome through the Thioredoxin-Interacting Protein (TXNIP) [12], an inhibitor of the ROS scavenger Thioredoxin, and promotes oxidative stress [13]. TXNIP links oxidative stress to inflammation, leading to cellular dysfunction and it is implicated in several diseases that are at risk for AD [14]. Although recent studies are suggesting a possible role of TXNIP in AD [15–18] there is no study investigating the effect of TXNIP in activating the microglial NLRP3 inflammasome in an AD contest.

Receptor for advanced glycation end products (RAGE) is a multi-ligand transmembrane receptor of the immunoglobulin superfamily and is the counterreceptor of proinflammatory ligands, such as advanced glycation endproducts (AGEs), S100s, HMGB1, and Aβ [19]. RAGE mediates Aβ uptake into the brain, leading to neurovascular inflammation that in turn leads to synaptotoxicity [19]. Studies carried out using RAGE^−/−^ mice demonstrated that RAGE contributes to AD pathophysiology [19]. RP-1, a RAGE antagonist peptide, diminishes Aβ plaque load and ameliorates memory impairment in an AD mouse model [20], confirming that RAGE is a possible therapeutic target for AD [19]. Inhibition of microglial RAGE rescues neuronal dysfunction in an AD mouse model, suggesting that it is triggering in microglia exerts a key role in AD [21]. Understanding how RAGE exerts its mechanism of action in microglia is crucial in order to develop new therapeutic strategies. RAGE promotes inflammation in microglia by activating the NLRP3 inflammasome [22]. We previously demonstrated that TXNIP is a downstream effector of RAGE in retinal endothelial cells and Schwann cells [23, 24]. In addition, activation of RAGE by AGEs promotes TXNIP expression in endothelial cells, leading to NLRP3 proinflammatory activity, which is blocked by a RAGE antagonist [25]. Considering the role of microglial RAGE and NLRP3 in AD, we explore the role of RAGE–TXNIP axis in both in vitro microglia and in vivo. We unveil TXNIP key mechanism in mediating Aβ-induced mitochondrial dysfunction and subsequent NLRP3 activation, which in turn promotes inflammation in AD. In detail, we show that RAGE–TXNIP axis is responsible for Aβ transport from the cell surface to mitochondria, leading to aberrant activation of the Dynamin-Related Protein 1 (Drp1), a key factor in mitochondria fission, which in turn participates in mitochondria dysfunction and subsequent NLRP3 activation. Such a cascade ultimately promotes the secretion of IL-1β and the activation of the pyroptosis-associated protein GSDMD.

## Material and methods

### Detailed Materials and Methods are available in the supplementary material

Animals; lentiviral tools and lentiviral transduction in primary microglia; stereotaxic surgery and viral injection; ELISA analysis of TXNIP protein level in mice hippocampus; primary microglia culture; immunohistochemistry; analysis of Iba1 in mice hippocampus; Aβ dimers preparation and labeling; western blot; immunofluorescence analysis; cell fractionation; co-immunoprecipitation experiments; RNA extraction and RT-qPCR; determination of intracellular and mitochondrial ROS levels; measurement of mitochondrial membrane potential; measurement of secreted IL-1β and caspase-1 activity; statistical analysis.

## Results

### Treatment with Verapamil or TXNIP silencing reduces brain inflammation in 5xFAD mice

**Fig. 1:**
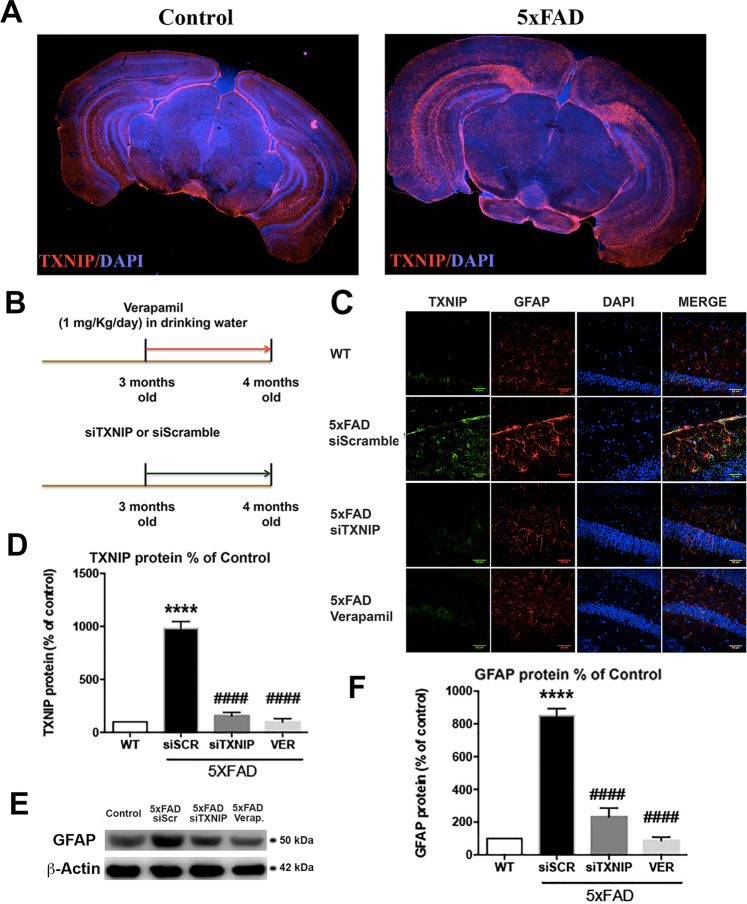
Treatment with Verapamil or TXNIP silencing reduce brain inflammation in 5xFAD mice. **A** IHC analysis of TXNIP expression in 4 m old control and 5XFAD mice **B** Schematic representation of Verapamil and siTXNIP treatment in 5xFAD mice. **C** IHC analysis of TXNIP (green) and GFAP (red) in 4 m old control and 5xFAD mice hippocampus treated as indicated (*n* = 4). **D** ELISA analysis of TXNIP in control and 5xFAD mice hippocampus treated as indicated. One-way ANOVA followed by Tukey’s multiple comparison test (*n* = 4, ****p < 0.0001 versus control, ^####^*p* < 0.0001 versus siScramble)**. E**, **F** Western blot analysis **E** and quantification **F** of GFAP in control and 5xFAD mice treated as indicated. One-way ANOVA followed by Tukey’s multiple comparison test (*n* = 3, ****p < 0.0001 versus control, ^####^*p* < 0.0001 versus siScramble). α-Actin was the load control.

TXNIP was overexpressed in the hippocampus of 4 months old 5xFAD mice compared with wild-type mice (wt) (Fig. 1A, C, D). Treatment with Verapamil, a calcium channels inhibitor that blocks TXNIP expression [26, 27], or TXNIP silencing (siTXNIP) (Fig. 1B), reduced TXNIP levels in 5xFAD mice, while siScramble injection had not any effect (Fig. 1C, D). It has been previously shown that TXNIP is overexpressed in neurons, astrocytes, and microglia in an AD mouse model [28]. As herein we were investigating the proinflammatory role of TXNIP, we analyzed the presence of TXNIP in astrocytes and microglia in the 5xFAD and wt mice. Glial Fibrillary Acidic Protein (GFAP) colocalized with TXNIP (Fig. 1C) and its levels were higher in 5xFAD mice, compared with wt mice (Fig. 1C, E, F). Verapamil and siTXNIP restored GFAP levels comparable to wt mice, while siScramble injection had no effect (Fig. 1C, E, F).

### TXNIP drives Aβ transport to mitochondria and promotes microglia activation

**Fig. 2:**
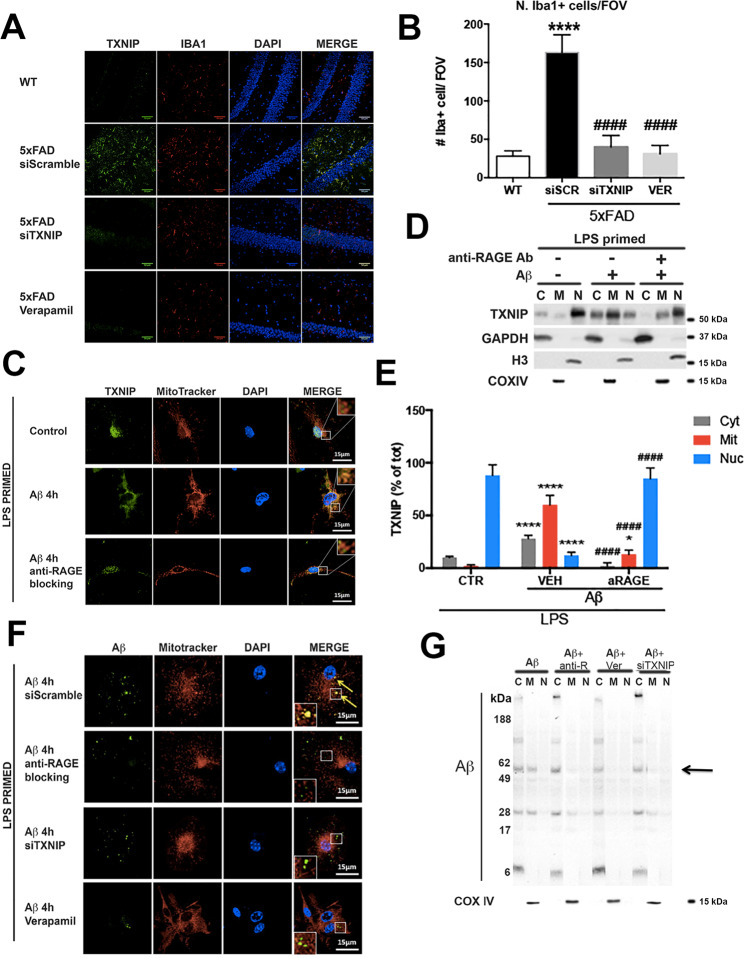
RAGE–TXNIP axis role in Aβ-promoted microglia dysfunction. **A** IHC analysis of TXNIP (green) and Iba1 (red) in 4 m old control and 5xFAD mice hippocampus treated as indicated. Nuclei are in blue (representative of *n* = 4). **B** Quantification of Iba1 positive microglia, expressed as cells per field of view (FOV) in WT and 5xFAD mice (Tg) treated as indicated. One-way ANOVA followed by Tukey’s multiple comparison test (****p < 0.0001 versus WT mice, ^####^*p* < 0.0001 versus 5xFAD mice treated with scrambled shRNA, *n* = 4). **C** IF analysis of TXNIP (green) and mitochondria (red), nuclei are in blue, in microglia treated as indicated (*n* = 5). **D**, **E** Subcellular fractionation, western blot analysis (**D**) and quantification (**E**) of TXNIP, GAPDH (cytoplasm), histone H3 (nucleus), Cox IV (mitochondria). One-way ANOVA followed by Tukey’s multiple comparison test (*n* = 5; ****p < 0.0001 versus control cells; ^####^*p* < 0.0001 versus cells treated with Aβ). **F** IF analysis of Aβ (green) and mitochondria (red) in microglia treated as indicated. Nuclei are in blue (representative of *n* = 5). **G** Subcellular fractionation and western blot of Aβ oligomers and Cox IV (representative of n = 4).

5xFAD mouse hippocampus showed an increase of Ionized calcium-binding adapter molecule 1 (Iba1) positive microglia compared to wt mice (Fig. 2A, B), that colocalized with TXNIP (Fig. 2A). Verapamil or siTXNIP treatment significantly lowered Iba1 positive microglia in 5xFAD hippocampus (Fig. 2A, B). Iba1 mRNA levels were increased in the hippocampus of 5xFAD mice compared to wt mice, and Verapamil or siTXNIP treatment restored Iba1 mRNA levels comparable to wt mice (Supplementary figure 1).

In LPS-primed microglia TXNIP was mostly localized into the nucleus (Fig. 2C). After 4 h of Aβ dimers exposure, TXNIP colocalized with mitochondria, whereas its nuclear localization was strongly diminished (Fig. 2C). Since TXNIP is regulated by RAGE [23–25], we analyzed the role of RAGE in TXNIP shuttling. Anti-RAGE-blocking antibody prevented Aβ-induced TXNIP translocation to mitochondria as we observed nuclear localization of TXNIP (Fig. 2C). Cellular fractionation confirmed that in control conditions ~90% of TXNIP was in the nuclear fraction (positive for histone H3), ~8% in the cytoplasm (positive for GAPDH), and ~2% in the mitochondrial fraction (positive for COX IV) (Fig. 2D, E). After Aβ dimers addition, ~60% of TXNIP was in the mitochondrial fraction and 30% in the cytoplasm, while the addition of anti-RAGE blocking antibody prevented the translocation of TXNIP to the mitochondrial fraction (Fig. 2D, E). Interestingly, Aβ colocalized with mitochondria 4 h after Aβ dimers addition (Fig. 2F). Anti-RAGE blocking antibody, siTXNIP or Verapamil, inhibited Aβ translocation to mitochondria, whereas did not prevent Aβ internalization (Fig. 2F). We detected Aβ oligomers with defined size (between 56 and 60 KDa) in the mitochondrial fraction after 4 h of Aβ dimers addition, and anti-RAGE blocking antibody, Verapamil or siTXNIP abolished the presence Aβ oligomers in the mitochondria fraction, without affecting Aβ internalization in the cytoplasmic fraction (Fig. 2G).

### TXNIP regulates Aβ interactions with Drp1 in LPS-primed primary microglia and Drp1 activation in vitro and in vivo

**Fig. 3:**
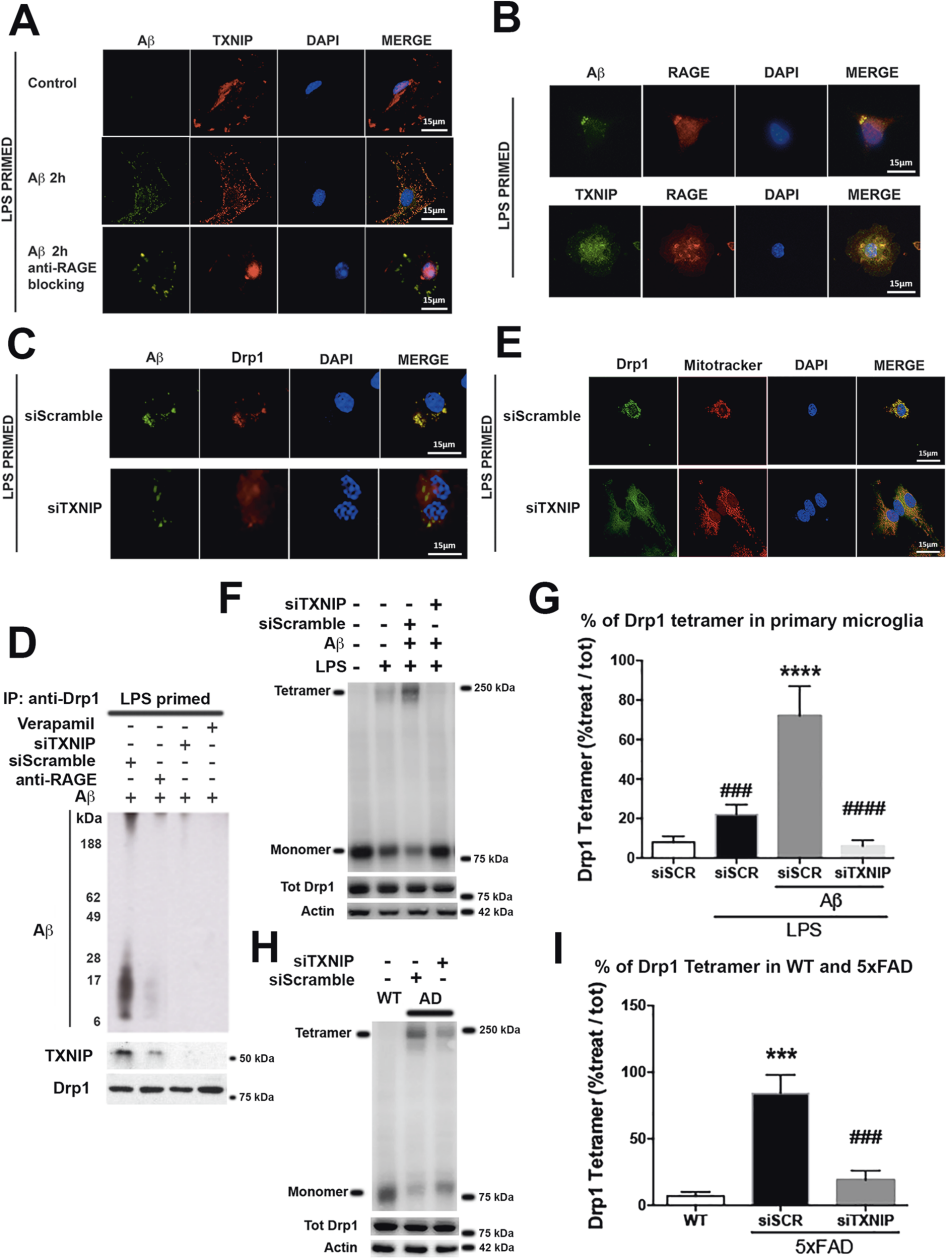
TXNIP is required for Drp1- Aβ interaction and Drp1 oligomerization in LPS-primed primary microglia and 5XFAD Tg mice. **A** IF analysis of Aβ (green) and TXNIP (red), nuclei are in blue (representative of *n* = 5). **B** IF analysis of Aβ (green), TXNIP (green), and RAGE (red), nuclei are in blue (representative of *n* = 5). **C** IF analysis of Aβ (green) and Drp1 (red), nuclei are in blue (representative of *n* = 3). **D** Western blot analysis of Aβ oligomers, TXNIP, and Drp1 after co-immunoprecipitation with an anti-Drp1 antibody (representative of *n* = 4). **E** IF analysis of Drp1 (green) and Mitochondria (red), in microglia, treated as indicated nuclei are in blue (representative of *n* = 3). **F**, **G** Western blot of native gel (**F**) and quantification (**G**) of Drp1 tetramer in LPS-primed primary microglia. Total Drp1 and actin were used to normalize. One-way ANOVA followed by Tukey’s multiple comparison test (*n* = 3; ****p < 0.0001 versus control; ^###^*p* < 0.001, ^####^*p* < 0.0001 versus siScramble). **H**, **I** Western blot of native gel (**H**) and quantification (**I**) of DRP1 tetramer in wt and 5xFAD mice treated as indicated. Total Drp1 and actin were used to normalize. One-way ANOVA followed by Tukey’s multiple comparison test (*n* = 3; ***p < 0.001 versus control; ^###^*p* < 0.001 versus siScramble).

Next, we analyzed the role of RAGE and TXNIP in Aβ transport from the cell surface to mitochondria. In control conditions, TXNIP was mostly localized into the nucleus, it was also evident a small fraction of TXNIP in the periphery, while, 2 h after Aβ dimers addition, TXNIP completely colocalized with Aβ and anti-RAGE blocking antibody prevented this colocalization (Fig. 3A). Notably, already 30 min after Aβ dimers addition, both Aβ and TXNIP colocalized with RAGE (Fig. 3B).

The interaction of Aβ and Drp1 is crucial for mitochondrial dysfunction in neurons [29]. Drp1 colocalized with Aβ 4 h after Aβ dimers addition and siTXNIP prevented their colocalization (Fig. 3C). In agreement, Aβ dimers and oligomers co-immunoprecipitated with Drp1 after 4 h of Aβ dimers addition (Fig. 3D). TXNIP also co-immunoprecipitated with Drp1 (Fig. 3D). Anti-RAGE blocking antibody strongly reduced the association of Aβ and TXNIP with Drp1, while Verapamil and siTXNIP treatment completely abolished these interactions (Fig. 3D). Drp1 colocalized with mitochondria 4 h following Aβ dimers treatment and siTXNIP prevented Drp1 translocation to mitochondria (Fig. 3E). Mitochondria fission is promoted by the formation of Drp1 tetramers [30]. The addition of LPS to primary microglia resulted in the formation of a small fraction of Drp1 tetramers that were significantly increased by 4 h treatment with Aβ dimers. siTXNIP completely prevented Drp1 oligomerization (Fig. 3F, G). Following 4 h of Aβ treatment, we observed mitochondrial fractionation that was reduced by siTXNIP (supplementary figure 2). We also detected the presence of Drp1 tetramers in the hippocampus of 5xFAD mice compared to wt mice that were significantly reduced by siTXNIP (Fig. 3H, I).

### RAGE–TXNIP axis mediates Aβ-induced mitochondrial alteration in LPS-primed primary microglia

**Fig. 4:**
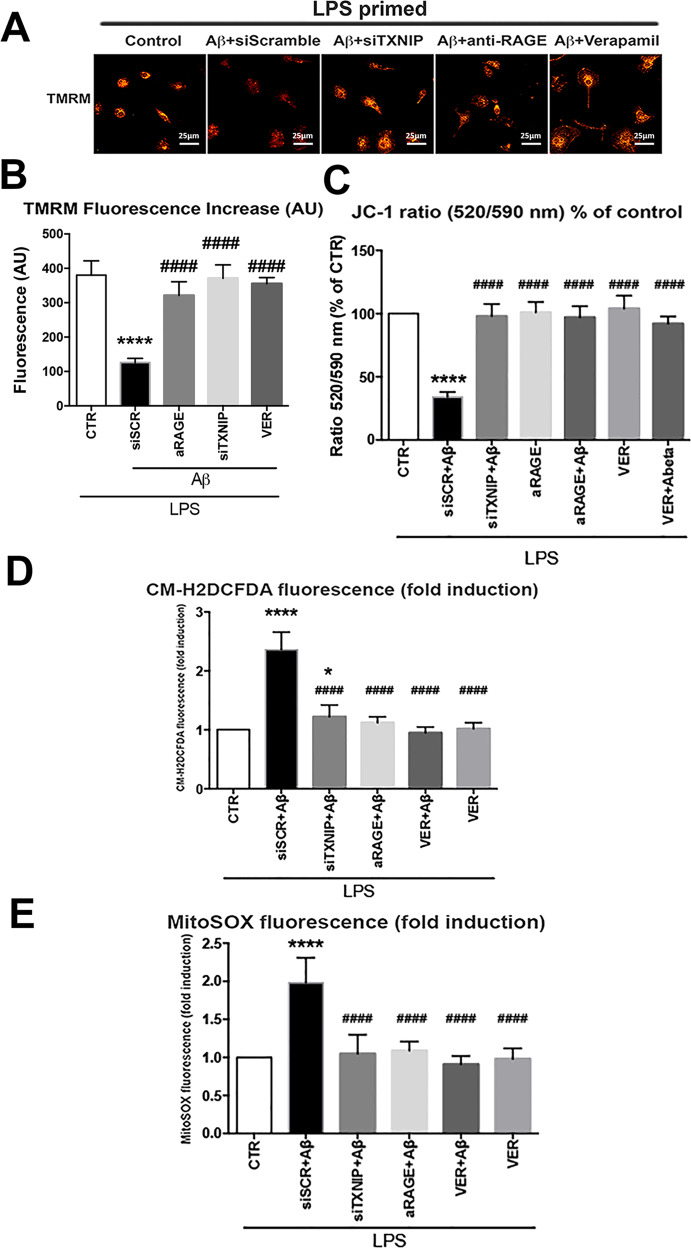
RAGE–TXNIP axis is implicated in Aβ-induced changes in mitochondrial membrane potential, ROS formation. **A**, **B** IF analysis (**A**) and quantification (**B**) of TMRM fluorescence in microglia treated as indicated. One-way ANOVA followed by Tukey’s multiple comparison test (*n* = 4; ****p < 0.0001 versus control; ^####^*p* < 0.0001 versus siScramble). **C** Quantification of JC-1 fluorescence ratio (520/590 nm) in microglia treated as indicated one-way ANOVA followed by Tukey’s multiple comparison test (*n* = 4; ****p < 0.0001 versus control; ^####^*p* < 0.0001 versus siScramble) **D** Quantification of CM-H2DCFDA fluorescence. One-way ANOVA followed by Tukey’s multiple comparison test (*n* = 12; ****p < 0.0001 versus control; ^####^*p* < 0.0001 versus siScramble). **E** Quantification of MytoSOX fluorescence. One-way ANOVA followed by Tukey’s multiple comparison test (*n* = 6; *p < 0.05, ****p < 0.0001 versus control; ^####^*p* < 0.0001 versus siScramble).

We investigated mitochondrial functionality by analyzing mitochondrial membrane depolarization (Δψm) using TMRM staining. Treatment for 4 h with Aβ dimers significantly reduced TMRM staining compared to control cells (Fig. 4A, B). Anti-RAGE blocking antibody, Verapamil, or siTXNIP treatment restored the TMRM fluorescence (Fig. 4A, B). Aβ dimers-induced reduction of the Δψm was confirmed by JC-1 staining (Fig. 4C). Treatment with Aβ dimers significantly induced both cytoplasmic (Fig. 4D) and mitochondrial ROS production (Fig. 4E). Verapamil, siTXNIP, or anti-RAGE blocking antibody reduced significantly Aβ-dependent cytoplasmic (Fig. 4D) and mitochondrial ROS production (Fig. 4E). Verapamil alone did not alter mitochondrial nor cytoplasmic ROS production (Fig. 4D, E).

### TXNIP is required for Aβ-induced NLRP3 inflammasome activation and IL-1β secretion in vitro and in vivo

**Fig. 5:**
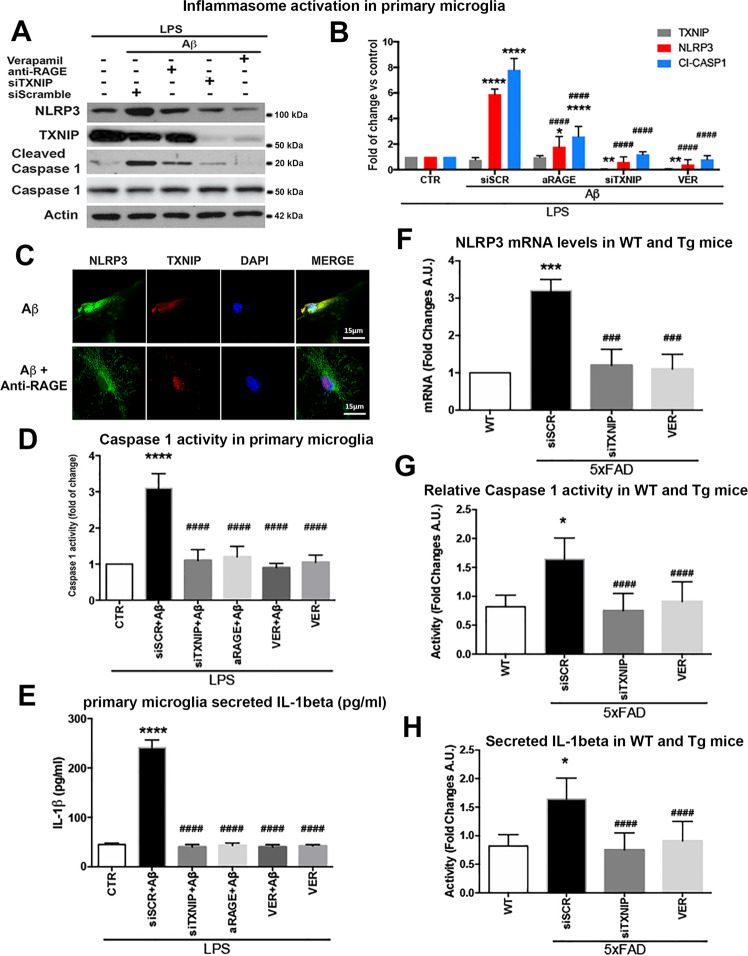
RAGE–TXNIP axis is implicated in Aβ-induced NLRPS inflammasome activation. **A**, **B** Western blot analysis (**A**) and quantification (**B**) of TXNIP, NLRP3, cleaved caspase-1 one-way ANOVA followed by Tukey’s multiple comparison test (*n* = 5; *p < 0.05, **p < 0.01, ****p < 0.0001 versus control; ^####^*p* < 0.0001 versus siScramble). Actin was used as loading control. **C** IF analysis of NLRP3 (green) and TXNIP (red), in microglia treated as indicated, nuclei are in blue (representative of *n* = 3). **D** Quantification of caspase-1 activity in microglia treated as indicated. One-way ANOVA followed by Tukey’s multiple comparison test (*n* = 5; ****p < 0.0001 versus control; ^####^*p* < 0.0001 versus siScramble). **E** ELISA of secreted Il-1β from the medium of microglia treated as indicated. One-way ANOVA followed by Tukey’s multiple comparison test (*n* = 6; ****p < 0.0001 versus control; ^####^*p* < 0.0001 versus siScramble). **F** RT-qPCR of GFAP mRNA in wt and 5xFAD (Tg) mice treated as indicated. One-way ANOVA followed by Tukey’s multiple comparison test (*n* = 3; ***p < 0.001 versus control; ^###^*p* < 0.001 versus siScramble). **G** Quantification of caspase-1 activity in wt and 5xFAD hippocampus of mice treated as indicated. One-way ANOVA followed by Tukey’s multiple comparison test (*n* = 4; *p < 0.05 versus control; ^####^*p* < 0.0001 versus siScramble). **H** ELISA of secreted IL-1β in wt and 5xFAD hippocampus of mice treated as indicated. One-way ANOVA followed by Tukey’s multiple comparison test (*n* = 4; *p* < 0.05 versus control; ^####^*p* < 0.0001 versus siScramble).

We next assessed the role of RAGE–TXNIP axis in the activation of the NLRP3 inflammasome complex and the subsequent caspase-1 activation and IL-1β secretion [31]. In LPS-primed microglia, 4 h of Aβ treatment significantly increased NLRP3 protein level and caspase-1 cleavage (Fig. 5A, B), while TXNIP was unaltered (Fig. 5A, B). Verapamil, siTXNIP, or anti-RAGE blocking antibody prevented Aβ-induced increment of NLRP3 and cleaved caspase-1, while anti-RAGE blocking antibody did not affect TXNIP protein levels (Fig. 5A, B). TXNIP and NLRP3 colocalized after 4 h of Aβ dimers addition, while anti-RAGE blocking antibody prevented their colocalization (Fig. 5C). Aβ dimers addition for 4 h significantly augmented caspase-1 activity that was inhibited by Verapamil, siTXNIP, or anti-RAGE blocking antibody (Fig. 5D). Similarly, Aβ dimers (6 h treatment) significantly induced IL-1β secretion that was completely prevented by Verapamil, siTXNIP, or anti-RAGE blocking antibody (Fig. 5E). Verapamil alone did not induce IL-1β secretion or caspase-1 activity (Fig. 5D, E). According to the data in vitro, NLRP3 mRNA expression significantly increased in the hippocampus of 4 m old 5xFAD mice compared to wt mice, siTXNIP or Verapamil restored NLRP3 mRNA levels, comparable to wt mice (Fig. 5F). Similarly, caspase-1 activity was strongly enhanced in the hippocampus of 4 m old 5xFAD mice compared to wt mice, while siTXNIP or Verapamil prevented this activation (Fig. 5G). The secreted IL-1β levels were significantly enhanced in the hippocampus of 4 m old 5xFAD mice compared to wt mice, while siTXNIP or Verapamil strongly decreased IL-1β release (Fig. 5H)

### Suppression of TXNIP blocks the cleavage of the pro-pyroptotic GSDMD

**Fig. 6:**
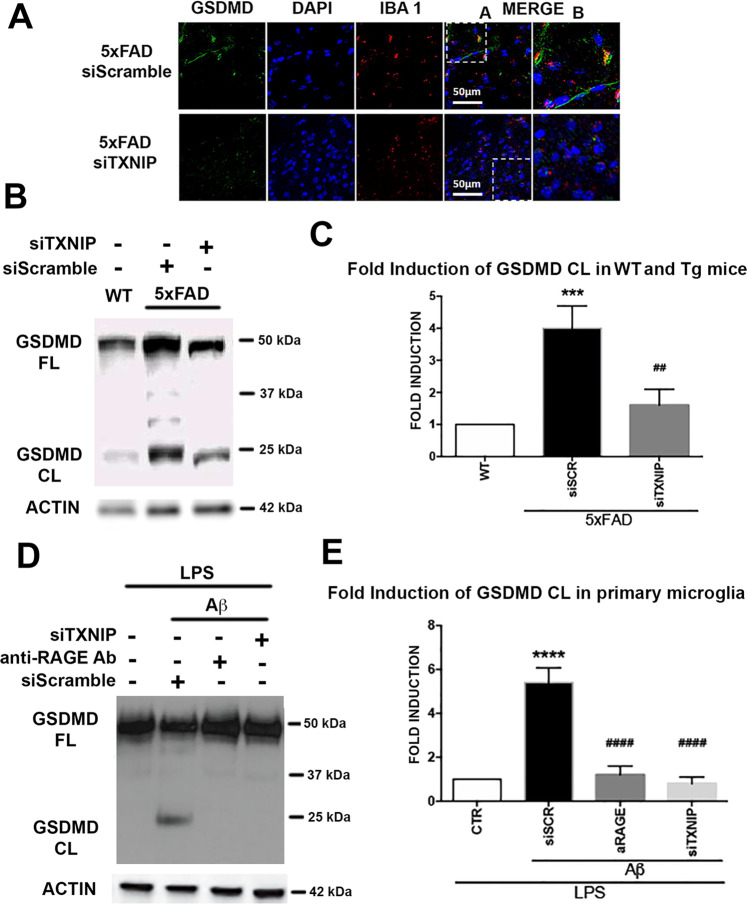
TXNIP silencing prevents GSDMD cleavage in vitro microglia and in vivo 5xFAD. **A** IHC analysis of GSDMDC1 (green) and Iba1 (red) in 4 m old control and 5xFAD mice hippocampus treated as indicated (*n* = 4). **B**, **C** Western blot of native gel (**B**) and quantification (**C**) of GSDMD cleavage in wt and 5xFAD mice hippocampus treated as indicated. Total GSDMD and actin were used to normalize. One-way ANOVA followed by Tukey’s multiple comparison test (*n* = 3; ***p < 0.001 versus control; ^##^*p* < 0.01 versus siScramble). **D**, **E** Western blot of native gel (**D**) and quantification (**E**) of GSDMD cleavage in LPS-primed primary microglia treated as indicated. Total GSDMD and actin were used to normalize. One-way ANOVA followed by Tukey’s multiple comparison test (*n* = 3; ****p < 0.0001 versus control; ^####^*p* < 0.0001 versus siScramble).

GSDMD colocalized with Iba1 positive cells in the hippocampus of 4 m old 5xFAD and siTXNIP prevented their colocalization (Fig. 6A). Cleaved GSDMD was increased in the hippocampus of 4 m old 5xFAD compared to wt mice and siTXNIP strongly reduces GSDMD cleavage (Fig. 6B, C). Similarly, the addition of Aβ dimers to LPS-primed primary microglia for 4 h resulted in GSDMD cleavage, which was prevented by siTXNIP or anti-RAGE-blocking antibody (Fig. 6D, E).

## Discussion

This study demonstrates for the first time that RAGE–TXNIP axis is required for Aβ transport to mitochondria, inducing NLRP3 inflammasome activation, secretion of IL-1β, and activation of the pyroptosis-associated protein GSDMD in microglia. In agreement, TXNIP inhibition is neuroprotective, while its overexpression is linked with damaging signals in neurons and endothelial cells [32]. Here we show, in agreement with other studies [15–17, 19, 32–34], that TXNIP is overexpressed in hippocampus of 5xFAD mice. Silencing of TXNIP in 5xFAD mice results in a significant reduction of GFAP protein, IL-1β release, GSDMD cleavage, and Iba1 positive cells in the hippocampus, showing a crucial role of TXNIP in promoting inflammation.

These data are confirmed by treating 5xFAD mice with Verapamil, an inhibitor of calcium channels, known as an efficient downregulator of TXNIP [27, 35]. Despite Verapamil being widely used for the treatment of high blood pressure, it has been recently proposed as a drug option for AD [36]. Indeed, Verapamil exerts neuroprotective effects in an AD rat model [37], reduces Aβ-induced neurotoxicity, and lowers Aβ1–40 oligomer formation [38]. On the other hand, here we show that Verapamil has a beneficial effect in our AD models mostly by blocking TXNIP expression, and its effects are comparable with data obtained by silencing TXNIP in vitro and in vivo. Remarkably, LPS-primed microglia treated with Verapamil alone do not show mitochondrial membrane potential alterations, nor enhanced mitochondrial or cytoplasmic ROS production, and do not have any effect on caspase-1 activity and IL-1β secretion. Thus, we exclude any other possible Verapamil effect unrelated to TXNIP expression on the NLRP3 cascade.

TXNIP is also associated with RAGE, a central player in immune response [39]. The binding of different ligands to RAGE on different cell types, triggers a different range of signaling pathways depending upon the cell phenotype [19, 23, 24, 40, 41]. Aβ is a ligand of RAGE [19], which mediates Aβ transport to mitochondria in cortical neurons [42, 43]. Using a primary microglia model primed with LPS [44], we demonstrate a functional interaction between TXNIP and Aβ. By immunofluorescence analysis and cell fractionation experiments, we show that TXNIP is localized mainly in the nucleus in primed microglia and translocated to mitochondria after exposure to Aβ dimers. It is known that oxidative stress promotes the shuttling of TXNIP from the nucleus to the mitochondria [45]. However, we demonstrate that RAGE plays a key role in TXNIP translocation to mitochondria induced by Aβ. Shortly after Aβ treatment, RAGE colocalizes with both Aβ and TXNIP. Remarkably, RAGE blocking prevents the translocation of Aβ to mitochondria, which is also blocked by Verapamil and TXNIP silencing. These data strongly suggest the involvement of RAGE and TXNIP in Aβ transport to mitochondria. Indeed, TXNIP belongs to the α-arrestin family involved in protein endocytosis [46, 47]. Notably, RAGE inhibition, Verapamil, or TXNIP silencing hamper the presence of defined Aβ oligomers in mitochondria, which are formed after treating the cells with Aβ dimers. We previously showed that toxic oligomers are formed after the internalization of Aβ dimers [48]. In particular, the 56mer Aβ oligomer, that we observe in our experiments, is known to have a strong toxic effect [49, 50]. These data suggest that RAGE–TXNIP axis promotes the formation of size-defined toxic oligomers that are translocated into mitochondria, further supporting the hypothesis that certain oligomers exert a more toxic effect.

Among several proteins that are involved in fission mitochondrial reactions, Drp1 is particularly relevant in neurodegenerative diseases. Drp1 drives mitochondrial fission within neurons [51, 52]. In the brain of AD patients, Drp1 directly interacts with Aβ monomers and oligomers, and their interaction increases with disease progression [53]. The interaction of Aβ with Drp1 is a crucial event for abnormal mitochondrial dynamics and mitochondrial dysfunction, finally contributing to synaptic damage in neurons [29, 54]. We confirm Drp1-Aβ interaction also in primed microglia. Indeed, Drp1 colocalizes with Aβ 4 h after Aβ addition and forms a protein complex together with Aβ oligomers. Noteworthy, TXNIP is also present in this complex and its silencing impedes Aβ-Drp1 colocalization. TXNIP silencing as well as inhibition of RAGE signaling completely abolishes the formation of the Aβ-Drp1 protein complex. Furthermore, 4 h Aβ dimers treatment results in the translocation of Drp1 to mitochondria, and this event is blocked by TXNIP silencing. Upon activation, Drp1 is recruited from the cytosol to the outer mitochondrial membrane, where it assembles by self-oligomerization to initiate mitochondrial division [30]. Treatment with Aβ leads to Drp1 oligomerization in oligodendrocytes [55]. Here we show that Aβ addition results in an enhanced Drp1 oligomerization also in primed microglia. Notably, TXNIP silencing prevents Drp1 oligomerization as well as blocks Drp1 translocation to mitochondria. We also observe that Aβ-induced enhanced Drp1 oligomerization and translocation to mitochondria in primed primary microglia results in enhanced mitochondria fragmentation, which is reduced by TXNIP silencing, showing that Aβ–Drp1 interaction leads to aberrant Drp1 activity and that TXNIP is essential for Aβ-Drp1 interaction. The hippocampus of 5xFAD mice shows an augmented Drp1 oligomerization compared to wt mice and TXNIP silencing strongly reduces Drp1 oligomerization. In agreement with our data, Drp1 oligomerization is enhanced in postmortem cortex samples from AD patients and in the corpus callosum of 5XFAD mice [55]. Our data corroborate the hypothesis that Aβ interaction with Drp1 leads to aberrant Drp1 activation in AD [54] and unveil the key role of TXNIP in this process.

Drp1 aberrant activation and Aβ transport to mitochondria are associated with mitochondrial dysfunction and altered mitochondrial membrane potential [30, 54]. According to previous studies [56], we demonstrate that Aβ induces mitochondrial dysfunction in primed microglia, as shown by reduced Δψm analyzed using 2 different markers: TMRM and JC-1, and increased oxidative stress both in mitochondria and in the cytoplasm. Reduced Δψm and enhanced mitochondrial ROS production were largely demonstrated in AD [57]. TXNIP silencing, Verapamil, or RAGE inhibition completely block Aβ -induced reduction of Δψm and oxidative stress, further supporting the role of RAGE–TXNIP axis in mediating mitochondrial damage due to Aβ, by mediating Αβ transport to mitochondria and interaction with Drp1.

Oxidative stress, mitochondrial dysfunction as well as aberrant Drp1 activation are implicated in the activation of the NLRP3 inflammasome [55, 58–61]. NLRP3 inflammasome activation is involved in several multifactorial diseases including AD [9, 62]. It has been shown that Aβ induces the activation of the NLRP3 inflammasome [63]. It is known that RAGE activates the NLRP3 inflammasome [64], as well as TXNIP, which promotes the NLRP3 assembly in microglia [65]. However, it has not yet been analyzed cooperation between RAGE, Aβ, and TXNIP in inducing the NLRP3 inflammasome. Upon activation, NLRP3 assembles a multi-protein platform that activates caspase-1, which drives the cleavage of pro-IL-1β and its secretion. Here we show that the Aβ−induced NLRP3 activation cascade is prevented in vitro by Verapamil, RAGE inhibition, or TXNIP silencing and in vivo by TXNIP silencing. Indeed, we show that in primed primary microglia Aβ-induced enhanced NLRP3 protein levels and caspase-1 cleavage are prevented by Verapamil, siTXNIP, or RAGE inhibition. TXNIP promotes NLRP3 assembly [65]. In agreement, a fraction of TXNIP colocalizes with NLRP3 after Aβ treatment, and RAGE inhibition prevents this colocalization. Verapamil, RAGE inhibition, or TXNIP silencing block Aβ-induced caspase-1 activity and IL-1β secretion in primed primary microglia. In 5xFAD mice, Verapamil or TXNIP silencing strongly reduces NLRP3 expression, caspase-1 activity, and secreted IL-1β. Altogether, these data show that RAGE–TXNIP axis drives NLRP3 inflammasome activation via Aβ.

It has previously been reported the presence of GSDMD in Iba1 positive cells in the corpus callosum of 5xFAD mice [55]. Moreover, the inhibition of NLRP3 inflammasome complex activation blocks the activation of caspase-1 and the subsequent cleavage of GSDMD in oligodendrocytes of 5xFAD mice [55]. Herein, we show an increased density of Iba1 positive cells also in the hippocampus of 5xFAD mice and the colocalization of GSDMD with Iba1 staining. TXNIP silencing reduces both Iba1 and GSDMD staining and prevents their colocalization. Notably, we show that TXNIP silencing blocks both in vitro and in vivo the cleavage of GSDMD. Although very recent studies suggest that TXNIP promotes pyroptosis in the nervous system through oxidative stress and NLRP3 pathway [66, 67], this is the first study providing a clear demonstration of the key role of TXNIP in GSDMD cleavage.

**Fig. 7:**
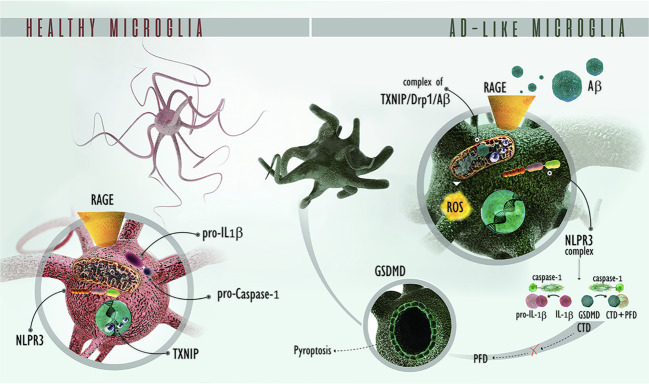
Schematic representation of the RAGE–TXNIP pathway implicated in microglia dysfunction in an AD contest. RAGE–TXNIP drives Aβ from the cell surface to mitochondria, leading to Drp1 oligomerization and translocation to mitochondria. These events lead to NLRP3 complex formation, resulting in caspase-1 activation, which in turn results in IL-1β secretion and GSDMD cleavage, producing CTD and PFD. The Pore Forming Domain (PFD) of cleaved GSDM assembles at the plasma membrane, inducing pyroptosis. *RAGE* receptor for advanced glycation endproducts. *TXNIP* thioredoxin-interacting protein. *AD* Alzheimer’s disease. *Aβ* Amyloid-beta peptide. *Drp1* Dynamin-related protein 1, *NLRP3* NOD-like receptor family, pyrin domain containing 3. *Pro-IL-1β* pro-interleukin 1 beta, *IL-1β* interleukin 1 beta, *GSDMD* gasdermin D, *CTD* C terminal domain (of GSDMD), *PFD* pore-forming domain.

In summary, we provide mechanistic evidence showing that Aβ detrimental effects require the activation of the RAGE–TXNIP endocytic pathway to drive Aβ into microglial mitochondria, which in turn promote NLRP3 inflammasome complex activation, IL-1β release, and GSDMD cleavage (Fig. 7). The recent knowledge about Drp1 role in promoting NLRP3 activation in innate immunity [67], strongly suggests that RAGE–TXNIP axis mediates the interaction between oligomeric Drp1 and Aβ followed by NLRP3 inflammasome complex activation. Our results strongly indicated that TXNIP is a druggable target of AD and this hypothesis is confirmed by Verapamil treatment. Since TXNIP, RAGE, Drp1 are implicated in several diseases of the neuronal system, our data open the way for understanding the pathological role of the RAGE–TXNIP axis in neurodegeneration.

### Reporting summary

Further information on research design is available in the Nature Research Reporting Summary linked to this article.

### Supplementary information

Supplementary material and methods

Supplementary Figure Legends

Supplementary Figure 1

Supplementary Figure 2

supplementary data

Reporting summary

## Data Availability

The data and materials included in this manuscript are available from the authors.
