# Correction to: RAGE-TXNIP axis drives inflammation in Alzheimer’s by targeting Aβ to mitochondria in microglia

**DOI:** 10.1038/s41419-022-04840-7

**Published:** 2022-04-19

**Authors:** Oualid Sbai, Mehdi Djelloul, Antonia Auletta, Alessandro Ieraci, Carlo Vascotto, L. Perrone

**Affiliations:** 1 Caminnov sas, Montpellier, France; 2 University Aix-Marseille, Marseille, France; 3 Department of Advanced Medical and Surgical Sciences, 2nd Division of Neurology, Center for Rare Diseases and InterUniversity Center for Research in Neurosciences, University of Campania Luigi Vanvitelli, Naples, Italy; 4 Department of Pharmaceutical Sciences, University of Milan, Milan, Italy; 5 Department of Medicine, University of Udine, Udine, Italy; 6 DKFZ, Department of Functional and Structural Genomics, Heidelberg, Germany; 7 University of Poitiers, Poitiers, France

**Keywords:** Cellular neuroscience, Inflammasome

Correction to: *Cell Death* and *Disease* 10.1038/s41419-022-04758-0, published online 04 April 2022

The original version of this article unfortunately contained a typo in the title. It was written: “AGE-TXNIP”, whereas the right title is “RAGE-TXNIP”. The authors apologize for the mistake. The original article has been corrected.

