# Supplementary material for: RAGE-TXNIP axis drives inflammation in Alzheimer’s by targeting Aβ to mitochondria in microglia
Source: Cell Death Dis. 2022 Apr 4;13(4):302. doi: 10.1038/s41419-022-04758-0 (PMC8980056; doi:10.1038/s41419-022-04758-0)
Supplement: Supplementary file 1 — Supplementary material and methods [file 41419_2022_4758_MOESM1_ESM.pdf]

SUPPLEMENTARY MATERIAL

We provide herein detailed methods.

MATERIAL and METHODS

Animals

Animal treatment and maintenance were performed in accordance with the guidelines published by European Communities Council Directive of November 24, 1986 (86/609/EEC). Animal experiments were approved by internal committees at the DKFZ Heidelberg, by Regierungspräsidium Karlsruhe (Germany), by the Ethical and Animal Care Committee N°84 COMETHEA of Poitou Charentes and by the French ministry (agreement number: 376–201 5072717461 531). An agreement was obtained from The High Council of Biotechnology for transgenic animals in 2011 and renewed in 2015 (agreement number: 2040). All efforts were made to minimize animal suffering and to reduce the number of mice used. The generation of 5XFAD mice has been described previously (1). These transgenic mice overexpress both mutant human APP (695) with the Swedish (K670N, M671L), Florida (I716V), and London (V717I) familial Alzheimer's Disease (FAD) mutations and human PS1 harboring two FAD mutations, M146L and L286V. Expression of both transgenes is regulated by neural-specific elements of the mouse *Thy1* promoter to drive overexpression in neurons.

The 5XFAD strain (B6/SJL genetic background) was maintained by crossing hemizygote transgenic mice with B6/SJL F1 breeders (Jackson Laboratories, Bar Harbor, Maine, USA). 5XFAD heterozygote transgenic mice were used for the experiments with non-transgenic wild-type (WT) littermate mice as controls. All transgenic and WT mice were bred in our animal facility, had access to food and water *ad libitum*, and were housed under a 12 h light-dark cycle at 22–24°C.

Verapamil (1 mg/kg/day) was added in the drinking water of 5xFAD mice of 2m of age (first formation of intracellular amyloid Aβ deposition) for 1 month (2). We previously demonstrated that this Verapamil was safe (2). Control wild type mice, untreated 5xFAD and Verapamil treated 5xFAD mice were sacrificed (animals received anesthesia, then sacrificed by cervical dislocation).

Lentiviral tools and lentiviral transduction in primary microglia

Custom MISSION-control shRNA Lentiviral transduction particles (scramble shRNA) and custom

MISSION-shRNA Lentiviral transduction particles targeting mouse TXNIP were purchased from Sigma-Aldrich (product ID TRCN0000182360): Sequence: CCGGGCAGAAGATCAGACCATCCATCTCGAGATGGATGGTCTGATCTTCTGCTTTTTTG. Functional validation of lentiviral tools was performed *in vitro* on primary microglia. We used a ration of virus to target cells (MOI)=50, which showed a high transduction efficiency as previously reported (3). Forty-eight hours later, cells were harvested and processed for TXNIP expression by western blot analysis.

Stereotaxic surgery and viral injections

Stereotaxic surgery to target the hippocampus for viral injection was performed under ketamine/xylazine anesthesia (4). Mice were injected into the hilus of the dentate gyrus of the hippocampus (−2.0 mm anterior–posterior, 1.3 mm medial–lateral, and −1.9 mm dorsal–ventral relative to the bregma) (5) with lentivirus particles (1x109 pfu/ml, 500 nl/side over a 10 min period, injection rate of 50 nl/min) (4). At the end of the experiment animals received anesthesia and then sacrificed by cervical dislocation.

ELISA analysis of TXNIP protein level in mice hippocampus

TXNIP protein amount in mice hippocampus was measured by using commercial sandwich Elisa kit (LifeSpan BioSciences, USA) according to manufacturer's instructions. Protein extracts were obtained as previously described (2).

Primary microglia culture

Primary microglia and astrocytes were obtained from 2-day-old CD1 mice brains. After removal of the meninges, the brains were dissociated into a single-cell suspension by trypsinization and mechanical disruption. The cells were seeded and grown at 37°C in a 5% CO2 humidified atmosphere in Dulbecco's modified Eagle medium (DMEM) containing 10% fetal calf serum (FCS), 2 mM L-glutamine, penicillin (100 U/mL), and streptomycin (100 μg/mL) (all from Invitrogen, Carlsbad, CA). The medium was replaced every 3 days. Microglia (90% pure) were separated by astrocytes after 1 week of co-culture and maintained in astrocytes-conditioned medium (DMEM, 10%FCS, 2 mM L-glutamine, 100 U/mL penicillin and 100 mg/mL streptomycin). After 1 day culture in medium containing 2% FCS, primary microglia were primed with Lipopolysaccharide (LPS) from

Escherichia coli serotype 055:B5 (Sigma-Aldrich) (100 ng/mL) for 6 h and then treated for different times with 3 μM Aβ dimers as previously described (2, 6). TXNIP expression were inhibited by adding Verapamil 1 μM (7, 8) 24h before and during Aβ dimers addition. Silencing of TXNIP by Custom MISSION-shRNA Lentiviral transduction particles targeting mouse TXNIP, as well as the control shScramble Lentiviral particles, were carried out 48h before Aβ dimers treatment. 24h before the addition of Aβ dimers, cells were shifted in DMEM containing 2% FCS 2 mM L-glutamine, penicillin (100 U/mL), and streptomycin (100 μg/mL). For the inhibition of RAGE activation, we added the anti-RAGE blocking antibody (RD System cat. N AF1179) 1h before and during Aβ dimers treatment, as we previously described (9-12).

Immunohistochemistry

Mice were deeply anesthetized (sodium pentobarbital, ip) and transcardially perfused (4% paraformaldehyde). Brains were extracted and post-fixed overnight in cold paraformaldehyde. Coronal brain sections (30 μm thick) were serially generated using a vibratome (Thermo Scientific HM650V) and stored at −20°C in 6-well plates containing 30% glycerol, 30% ethylene glycol in 0.05M PBS until processing. After washing in PBS, floating sections were incubated for 1h at room temperature (RT) with blocking solution (3% BSA, 0.1% Triton X-100 in PBS) and overnight at 4°C with the following primary antibodies: rabbit polyclonal anti-GFAP (1:400, Dako), mouse monoclonal anti-TXNIP (1:100, MBL, clone JY2), rabbit polyclonal anti-Iba1 (1:200, FujiFilm Wako), anti-GSDMDC1 (1:500, Novus). Slices were rinsed 5 min in PBS and incubated for 90 min at RT with cross-adsorbed Alexafluor 488- or 594-conjugated anti-rabbit or anti-mouse secondary antibodies (1:500, Jackson Immunoresearch). After 3 washes in PBS, slides were counter-stained with 0.5 ng/ml Hoechst blue (Sigma-Aldrich) for 30 min at RT and mounted with ProLong Gold Antifade reagent. Confocal image acquisition was performed on a Zeiss 510 laser-scanning microscope with 63× oil immersion objectives. Images were analyzed with the software Zen (Zeiss) as previously described (2, 11, 13). Images of large brain sections were acquired using a Zeiss inverted Axio Observer microscope (Zeiss, Jena, Germany) equipped with DAPI, FITC, and Rhodamine epifluorescence filters. These large images were obtained using the mosaic mode of the Axiovision software (Zeiss).

Analysis of Iba1 in mice hippocampus

Iba1 positive cells were automatically counted by using the Bitplane Imaris 7.5 software. Four comparable serial histological sections along the hippocampus were selected for analysis, for each mouse, by an operator blinded to the treatment. Images of the hippocampus were taken at 20X objective, at multiple z-planes, and total number of microglia counts, and volumes were automatically analyzed by the Bitplane Imaris 7.5 spots or surfaces modules, respectively.

Aβ dimers preparation and labeling

$A\beta_{1-42}$ was prepared as previously described (6): Aβ peptides were dissolved in 1,1,1,3,3,3-Hexafluoro-2-propanol (HFIP) in order to obtain 100 μM concentration. Aβ dissolved in HFIP was dried and dissolved in 100 μl NaOH 50 mM. Then, 0.9 ml of PBS was added. The solutions of $A\beta_{1-42}$ was maintained 2h on ice. For immunofluorescence analysis, the same protocol was used in order to label $A\beta_{1-42}$ with Fluorescein-5-isothiocyanate (FITC; Sigma Aldrich) for 2h on ice according to the manufacturer instructions. Following a centrifugation for 30 seconds, the peptides were filtered using a 0.2 µm filter. Both unlabeled and FITC labeled $A\beta_{1-42}$ were subjected to a chromatographic separation using Fluorescent Dye Removal Column (Thermo Fisher) according to the protocol provided by the manufacturer. This procedure allowed the purification of $A\beta_{1-42}$ dimers (6). Finally, the peptides were added to the cells.

Cell Fractionation

Microglia, seeded at a density of $5X10^6$ cells in 100 mm dishes, were treated as described above. Cytoplasmic, mitochondrial and nuclear fractions were obtained using a Cell Fractionation Kit-Standard (Abcam, product ab109719) according to the manufacturer instruction.

Co-Immunoprecipitation experiments

Microglia, seeded at a density of $5X10^6$ cells in 100 mm dishes, were treated as described above. Cells were lysed in 0,25 ml/dish of ice-cold extraction buffer (Hepes 20 mM pH 7,9; Nonidet-P40 1%; Deoxycholate-Na 0,25%; NaCl 150 mM; EDTA 1mM, EGTA 1mM; 1 mm dithiothreitol; phenylmethylsulfonyl fluoride 1 mM; Leupeptin 1 μg/ml; Aprotinin 1 μg/ml; Pepstatin1 μg/ml; Na_3VO_4 1 mM; NaF 1 mM). Protein extracts were centrifuged at 14.000 g for 15 min at 4°C. Supernatants were transferred in a new tube and diluted by adding 0,55 ml of ice-cold Co-IP solution (Hepes 20

mM pH 7,9; NaCl 150 mM; EDTA 1mM, EGTA 1mM; 1 mm dithiothreitol; phenylmethylsulfonyl fluoride 1 mM; Leupeptin 1 μg/ml; Aprotinin 1 μg/ml; Pepstatin1 μg/ml; Na_3VO_4 1 mM; NaF 1 mM). Protein A/G-Sepharose beads were washed twice with PBS containing 0,1% BSA, then dissolved in ice-cold Co-IP solution in order to obtain a 5X concentration of beads. Diluted protein extracts were pre-cleaned by adding 0,1 ml of ice-cold 5x Protein A/G-Sepharose beads solution and incubated 15 min at 4°C with gentle agitation. Beads were precipitated by centrifugation at 3000 rpm in a microfuge for 2 min, the supernatants were recovered in a new tube and immunoprecipitated by adding 2 μg of rabbit polyclonal anti-Drp1 (Sigma) 5 h at 4°C with gentle shacking, followed by addition of 0,1 ml of 5x Protein A/G-Sepharose beads solution and additional incubation for 2h at 4°C with gentle shacking. Beads were washed 3 times with ice-cold Co-IP buffer and once with ice-cold PBS. The pellets were resuspended in electrophoresis SDS sample buffer, boiled for 5 min at 95°C and analyzed by SDS PAGE electrophoresis and western blotting as described below.

Western blot

For analysis of proteins from brain tissues: after NaCl transcardial perfusion, the HPC were microdissected and snap-frozen for biochemical assays. Each sample was homogenized in 25% w/v of 50 mM Tris-HCl pH 7.5 buffer containing 150 mM NaCl, 2 mM EDTA, 1% Triton X-100, 1% SDS and proteinase inhibitor cocktail (Millipore, Molsheim, France) and centrifuged at 10,000*g* for 20 min. Protein concentrations were determined using the Lowry method (Bio-Rad, Hercules CA, USA). Total protein extracts from cells were obtained using RIPA buffer as previously described (2, 6, 12). After boiling, aliquots containing equal amounts of protein (30 μg) were loaded in Laemmli buffer and separated by 8% sodium dodecyl sulphate (SDS) polyacrylamide (Bio-Rad) gel electrophoresis (PAGE) using a MiniBlot system (Bio-Rad). For the analysis of Aβ oligomers, proteins were separated on a gradient 4-20% SDS-PAGE as previously described (6). Extracts without SDS and native gel electrophoresis were performed as previously described (14).

Western blotting was performed as previously described (9-11). Proteins were transferred onto nitrocellulose membranes (Amersham Biosciences) in transfer buffer (25 mM Tris, 192 mM glycine, 20% ethanol). Membranes were incubated overnight in blocking buffer at RT and then probed with the following primary antibodies, diluted in blocking buffer: mouse monoclonal anti-TXNIP (1:1000

in PBS 1% fat free milk, MBL, clone JY2); rabbit polyclonal anti-GFAP (1:2000 in PBS 5% fat free milk, Dako), rabbit polyclonal anti-IL-1β (1:2000 in PBS 5% fat free milk, Abcam); mouse monoclonal anti-actin (1:3000 in PBS 5% fat free milk, Sigma); rabbit polyclonal anti-Drp1 (1:2000 in PBS 5% fat free milk, Sigma); rabbit polyclonal anti-GAPDH (1:2000 in PBS 5% fat free milk, Sigma); rabbit polyclonal anti-histone H3 (1:2000 in PBS 5% fat free milk, Abcam ab1791); mouse monoclonal anti-Cox IV (1:2000 in PBS 5% fat free milk, Abcam ab14744); mouse monoclonal anti-Aβ (1:2000 in 5% BSA, Sigma, clone AB10); rabbit polyclonal anti-NLRP3 (1:2000 in PBS 5% fat free milk, PA5-79740 Invitrogen, TermoFisher); rabbit polyclonal anti-caspase-1 (1:2000 in PBS 5% fat free milk, PA5-87536 Invitrogen, TermoFisher), anti-GSDMD (1:2000, Novus). After incubation with primary antibodies, membranes were incubated with a horseradish-peroxidase conjugated secondary antibody (Jackson Immunoresearch, West Grove PA, USA). Finally, proteins were detected using a chemiluminescence kit (Roche Diagnostics). The signal was detected with the Odyssey CLx scanner (Li-Cor Bioscience) and densitometric analysis was performed with ImageStudio software (Li-Cor Bioscience). We also employed autoradigraphic films that were digitized using GeneTools software (Syngen) and ODs of the bands were assessed using scion image software (Scion Corporation MA, USA) as previously described (2, 15).

For Drp1 oligomerization, extracts were omogenized in 20 mM Hepes pH 7,5, 10 mM KCl, 1,5 mM $MgCl_2$, protease inhibitors. Samples were diluted in Laemly without SDS and DTT and subjected to electrophoresis in a native 10% acrylamide gel, then subjected to western blot as described above.

Immunofluorescence analysis

FITC-labeled Aβ dimers (see above) were incubated with primary microglia at 37°C at 3 μM final concentration for the time indicated in the text. MitoTracker (dilution 1:2000 in medium w/o serum) was added 30 minutes before cell fixation. Cells were fixed with 4% PFA in PBS. After washing in PBS, cells were permeabilized with 0.1% Triton X-100 in PBS for 5 minutes at RT. Following a wash in PBS, cells were incubated 1h at RT with blocking solution (3% BSA in PBS) and overnight at 4°C with the following primary antibodies diluted in blocking solution: mouse monoclonal anti-TXNIP (1:100, MBL, clone JY2), rabbit polyclonal anti-Drp1 (1:200, Sigma), rabbit polyclonal anti-NLRP3 (1:200, Invitrogen TermoFisher), rabbit polyclonal anti-RAGE (1:200, Santa Cruz). Then, cells were

rinsed (3-5 min) in PBS and incubated for 60 min at RT with cross-adsorbed Alexafluor 488- or 594-conjugated anti-rabbit or anti-goat secondary antibodies (1:500, Jackson Immunoresearch, West Grove, PA, USA) in dark conditions. Cells were washed twice with PBS and once with H_2O_2 and mounted in ProLong Gold Antifade reagent (Invitrogen). Inhibition of Aβ-RAGE interaction were performed by adding an anti-rat RAGE blocking antibody 5 minutes before and during Aβ addition, as previously described (9, 11, 12). For TXNIP silencing, cells were treated with shLentiviral vectors 48h before Aβ addition. Analysis of immunofluorescence was performed with a Zeiss LSM 510 confocal laser-scanning microscope. Images were analyzed with the software Zen (Zeiss) as previously described (2, 10, 11). Samples were visualized with an inverted microscope using a 60 oil-immersion lens, magnified two times and scanned sequentially to maximize signal separation. DAPI, AlexaFluor 488 and AlexaFluor 594 fluorescence were excited with a 405 nm blue laser diode, 488 nm argon blue and 543 nm helium neon laser, respectively. Emissions were separated with 430–460 nm, 505–525 nm, and 610 nm barrier filters.

RNA extraction and RT-qPCR

Brains were removed and the hippocampi were dissected and frozen. RNA extraction and RT-qPCR were performed as previously described (16). Oligonucleotide primer specific for mouse NLRP3 (forward: *5′-TGC TCT TCA CTG CTA TCA AGC CCT-3′*, reverse: *5′-ACA AGC CTT TGC TCC AGA CCC TAT-3′*). Oligonucleotide primers specific for mouse Iba1 (forward: *5′*-GGATTTGCAGGGAGG AAAAG*3′*, reverse, *5′*-TGGGATCATCGAGGA ATTG*3′*)

Determination of intracellular and mitochondrial ROS levels

Cells were plated on 96-wells plates and intracellular ROS levels were determined as previously described (17). The day before experiments, primary microglial cells were cultured in medium containing 2% FCS (Gibco) and maintained in low serum condition for 16h. The day of the experiments, cells were washed with PBS and incubated in 5% CO_2/95% air at 37°C for 6h in DMEM without FCS. Cells were washed twice with DMEM. Cells were treated with Aβ dimers (3 μM) for 4h at 37°C. Intracellular ROS levels were monitored by using the fluorescent dye 2',7'-dichlorodihydrofluorescein diacetate (H2DCFDA; Molecular-Probes, Invitrogen) as previously described (2, 18). After treatment, cells were washed twice and incubated in DMEM containing

H2DCFDA (20 μM). After 30 min incubation at 37°C, cells were washed with PBS and fluorescence intensity was measured in a Fluoro plate reader (FLX800 Biotek Instruments, software KL4) with the excitation and emission wavelengths at (485±20 nm 525±20 nm) respectively. The cell-permeant H2DCFDA is converted into a no fluorescent polar derivative (H2DCF) by cellular esterases after incorporation into cells. H2DCF is oxidized rapidly to the highly fluorescent 2',7'-dichlorofluorescein (DCF) in the presence of intracellular ROS. For mitochondrial ROS determination we employed MitoSox red superoxide indicator (Invitrogen, TermoFisher). Microglia was plated in 96 wells plates and treated as we described above for intracellular ROS determination. After treatment, cells were washed twice and incubated 15 min in DMEM containing MitoSox according to the manufacturer instruction. Fluorescence intensity was measured in a Fluoro plate reader (FLX800 Biotek Instruments, software KL4).

The results obtained are the average of three independent experiments each of them performed with 4 replicates for each point analyzed (n=12 and three independent experiments).

Measurement of mitochondrial membrane potential

Cells were plated near confluence on 24 mm coverslips, treated as described above and stained with Tetramethylrhodamine, methyl ester (*TMRM*) according to the manufacturer instruction (TMRM assay kit, Abcam ab228569). Confocal images were acquired with a Zeiss LSM 510 confocal microscope equipped with a plan-Apochromat 63× oil immersion objective. The 543-nm excitation wavelength was provided by a HeNe laser source. All images were obtained at a 5% laser potency and with a pinhole diameter of 1 airy unit as suggested by the manufacturer. Amplifier and detector optimizing parameters were maintained constant for all the experiments. A first set of images was obtained before stimulation with the different agonists; after addition of the stimulants, further images were acquired at 3 min intervals for 30 min. To accurately measure fluorescence emission from the entire mitochondrial network, a single 3D projection of confocal images on the *z*-axis was obtained by means of LSM examiner software (Carl Zeiss, Arese, Italy). Fluorescence was then quantitated and corrected for background emission with the cell imaging software MetaMorph 2.3 (Universal Imaging). Data were acquired from 10 to 15 cells per coverslip. An average of 10 coverslips were analyzed for each experimental condition.

To confirm the data obtained with TMRM, cells were plated on 96 well near confluence, treated as described above and stained with JC-1 according to the manufacturer instructions (MitoProbe JC-1 Assay kit, Invitrogen). Fluorescence intensity was measured in a Fluoro plate reader (FLX800 Biotek Instruments, software KL4). The ratio of JC-1 aggregates (red fluorescence, emission 585-615 nm) to monomers (green fluorescence, emission 515-545 nm) was measured. Mitochondrial depolarization was indicated by a decrement in the ratio of red/green fluorescence intensity. Fluorescence intensity was measured in a Fluoro plate reader (FLX800 Biotek Instruments, software KL4).

Measurement of secreted IL-1β and caspase-1 activity

Microglia, seeded at a density of $1X10^5$ cells/well in 24-well plates, were treated as described above. Supernatants were collected and levels of IL-1β in culture supernatant were determined by ELISA according to the manufacturer's instruction (BD Biosciences). The measurement of IL-1β in extracts from mice hippocampus was performed using the same ELISA kit according to the manufacturer's instruction (BD Biosciences). Protein extracts from mice hippocampus were obtained as previously described (2).

Microglia, seeded at a density of $1X0^6$ cells and treated as described above, were measured for caspase-1 activity according to the manufacturer's instruction (Abcam, Fluorimetric kit).

Statistical Analysis

Statistical analyses were performed using GraphPad6 (GraphPad Software Inc., USA). Graphs were presented as the mean value ± Standard Deviation (S.D.). All data were analyzed by a one-way ANOVA followed by Tukey's multiple comparison test when appropriated. Differences were considered significant for a p-value <0.05.

REFERENCES

1. Oakley H, Cole SL, Logan S, Maus E, Shao P, Craft J, et al. Intraneuronal beta-amyloid aggregates, neurodegeneration, and neuron loss in transgenic mice with five familial Alzheimer's disease mutations: potential factors in amyloid plaque formation. J Nerosci. 2006;26:10129-40.

2. Melone MAB, Dato C, Paladino S, Coppola C, Trebini C, Giordana M, et al. Verapamil Inhibits Ser202/Thr205 Phosphorylation of Tau by Blocking TXNIP/ROS/p38 MAPK Pathway. Pharm Res. 2018;35:44.

3. Maes ME, Colombo G, Schulz R, Siegert S. Targeting microglia with lentivirus and AAV: Recent advances and remaining challenges. Neurosci Let. 2019;707:134310.

4. Blouet C, Schwartz GJ. Nutrient-sensing hypothalamic TXNIP links nutrient excess to energy imbalance in mice. J Neurosci. 2011;31:6019-27.

5. Jeon SG, Kang M, Kim YS, Kim DH, Nam DW, Song EJ, et al. Intrahippocampal injection of a lentiviral vector expressing neurogranin enhances cognitive function in 5XFAD mice. Exp Mol Med. 2018;50:e461.

6. Mazarguil H, Leveque C, Bartnik D, Fantini J, Gouget T, Funke SA, et al. A synthetic amino acid substitution of Tyr10 in Aβ peptide sequence yields a dominant negative variant in amyloidogenesis. Aging Cell. 2012;11(3):530-41.

7. Chen J, Cha-Molstad H, Szabo A, Shalev A. Diabetes induces and calcium channel blockers prevent cardiac expression of proapoptotic thioredoxin-interacting protein. Am J Physiol Endocrinol Metab. 2009;296:E1133-E9.

8. Liu Y, Lo YC, Qian L, Crews FT, Wilson B, Chen HL, et al. Verapamil protects dopaminergic neuron damage through a novel anti-inflammatory mechanism by inhibition of microglial activation. Neuropharmacology. 2011;60:373-80.

9. Perrone L, Devi TS, Hosoya KC, Terasaki T, Singh LP. Thioredoxin Interacting Protein (TXNIP) induces inflammation through chromatin modification in retinal capillary endothelial cells under diabetic conditions. J Cell Physiol. 2009;221:262-72

10. Perrone L, Peluso G, Melone MAB. RAGE recycles at the plasma membrane in S100B secretory vesicles and promotes Schwann cells morphological changes. J Cell Physiol. 2008;217:60-71.

11. Sbai O, Devi TS, Melone MAB, Feron F, Khrestchatisky M, Singh LP, et al. RAGE-TXNIP axis is required for S100B-promoted Schwann cell migration, fibronectin expression and cytokine secretion. J Cell Sci. 2010;123:4332-9.

12. Vincent A, Perrone L, Sullivan K, Backus C, Sastry A, Lastoskie C, et al. Receptor for advanced glycation end products activation injures primary sensory neurons via oxidative stress. Endocrinology. 2007;148:548-58.

13. Perrone L, Sbai O, Nawroth PP, Bierhaus A. The Complexity of Sporadic Alzheimer's Disease Pathogenesis: The Role of RAGE as Therapeutic Target to Promote Neuroprotection by Inhibiting Neurovascular Dysfunction. Int J Alzheimers Dis. 2012;2012:734956-66.

14. Zhang X, Wang R, Hu D, Sun X, Fujioka H, Lundberg K, et al. Oligodendroglial glycolytic stress triggers inflammasome activation and neuropathology in Alzheimer's disease. Sci Adv. 2020;6:eabb8680.

15. Perrone L, Paladino S, Mazzone M, Nitsch L, Gulisano M, Zurzolo C. Functional interaction between p75NTR and TrkA: the endocytic trafficking of p75NTR is driven by TrkA and regulates TrkA-mediated signaling. Biochem J. 2005;385:233-41.

16. Perrone L, Devi TS, Hosoya KC, Terasaki T, Singh LP. Inhibition of TXNIP Expression In Vivo Blocks Early Pathologies of Diabetic Retinopathy. Cell Death Dis. 2010;e65:doi:10.1038/cddis.2010.42

17. Pines A, Perrone L, Bivi N, Romanello M, Damante G, Gulisano M, et al. Activation of APE1/Ref-1 is dependent on reactive oxygen species generated after purinergic receptor stimulation by ATP. Nucl Acid Res. 2005;33:4379-94.

18. Perrone L, Mothes E, Vignes M, Mockel A, Figueroa C, Miquel MC, et al. Copper transfer from Cu-Abeta to human serum albumin inhibits aggregation, radical production and reduces Abeta toxicity. Chembiochem. 2010;11:110-8.
