# Supplementary material for: RAGE-TXNIP axis drives inflammation in Alzheimer’s by targeting Aβ to mitochondria in microglia
Source: Cell Death Dis. 2022 Apr 4;13(4):302. doi: 10.1038/s41419-022-04758-0 (PMC8980056; doi:10.1038/s41419-022-04758-0)
Supplement: Supplementary file 2 — Supplementary Figure Legends [file 41419_2022_4758_MOESM2_ESM.pdf]

**SUPPLEMENTARY FIGURE LEGENDS**

**Supplementary Figure 1. Verapamil and siTXNIP lower Iba1 expression in the hippocampus of 5xFAD mice.** RT-qPCR of Iba1 hippocampal mRNA in wt and 5xFAD mice treated as indicated. One-way ANOVA followed by Tukey's multiple comparison test (n=5; $^{****}p<0.0001$ versus WT mice, $^{\#\#\#\#}p<0.0001$ versus 5xFAD mice treated with scramble shRNA)

**Supplementary Figure 2. TXNIP silencing ameliorates Aβ-induced mitochondria fractionation in primed primary microglia.** IHC of mitochondria using Mitotracker red. Nuclei are stained in blue with DAPI. Magnification of mitochondria are indicated with a square. Data are representative of 3 independent experiments.
