# Supplementary figures and images for: RAGE-TXNIP axis drives inflammation in Alzheimer’s by targeting Aβ to mitochondria in microglia
Source: Cell Death Dis. 2022 Apr 4;13(4):302. doi: 10.1038/s41419-022-04758-0 (PMC8980056; doi:10.1038/s41419-022-04758-0)

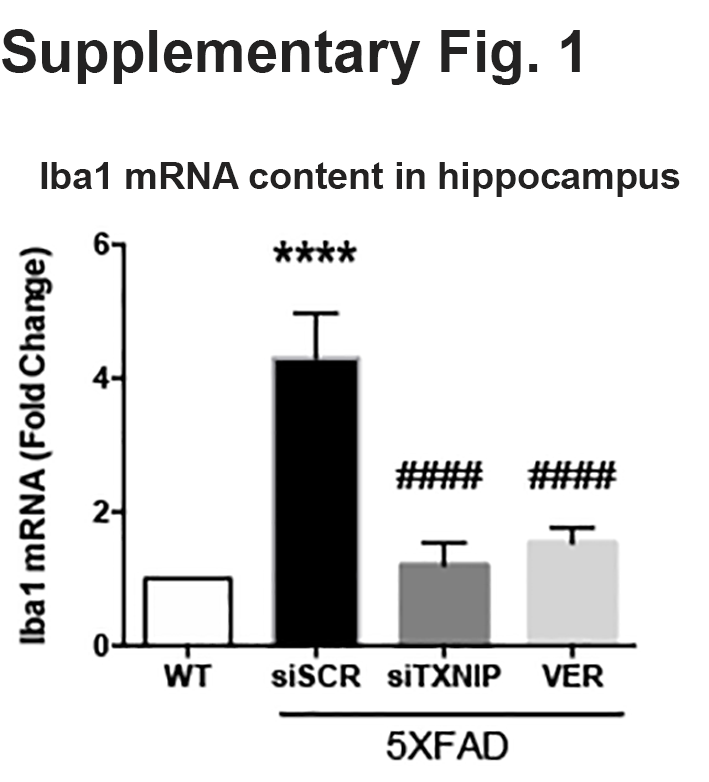

Supplement: Supplementary file 3 — Supplementary Figure 1 [file 41419_2022_4758_MOESM3_ESM.tif]

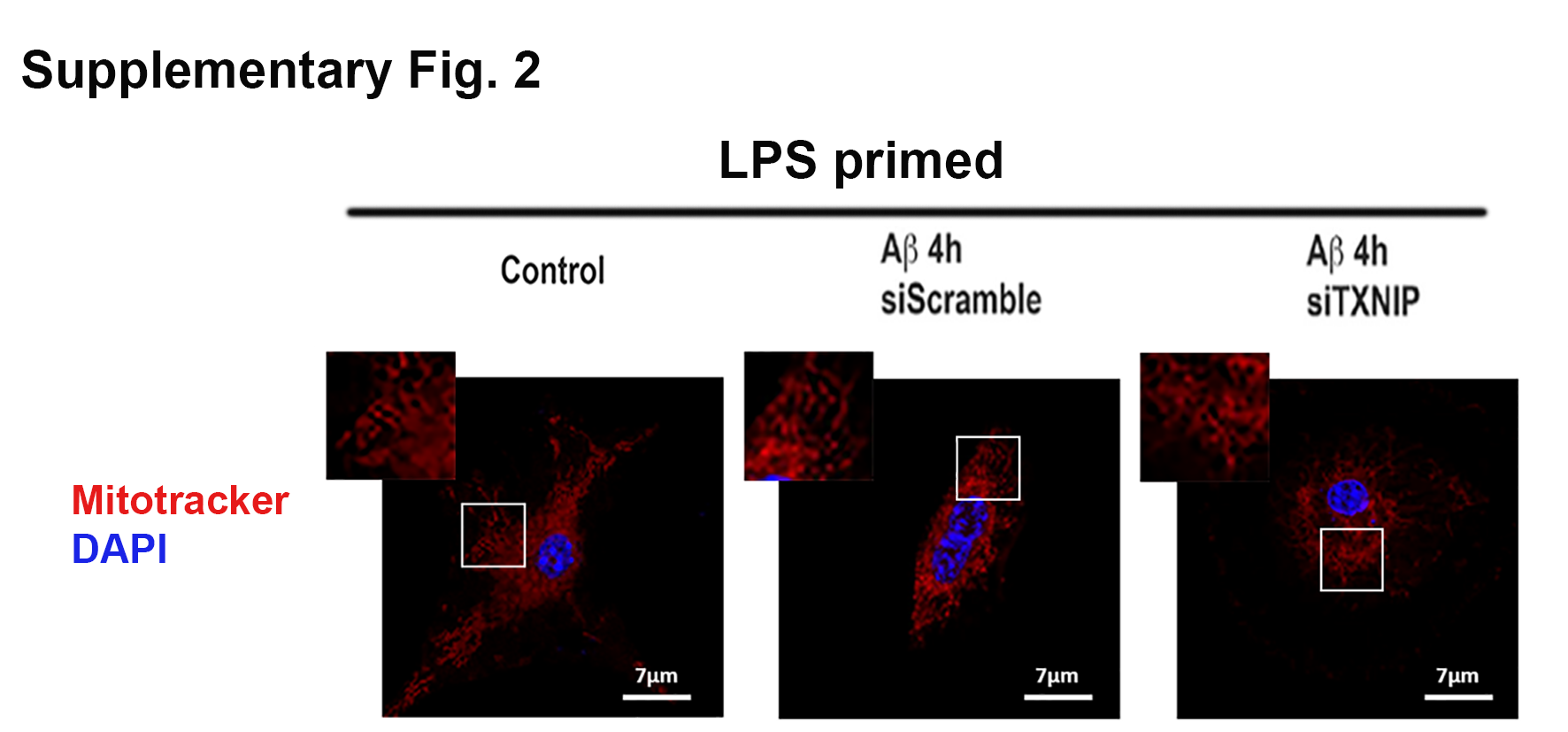

Supplement: Supplementary file 4 — Supplementary Figure 2 [file 41419_2022_4758_MOESM4_ESM.tif]

Fig. 1E

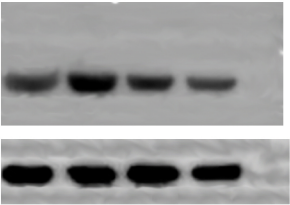

Fig. 2G

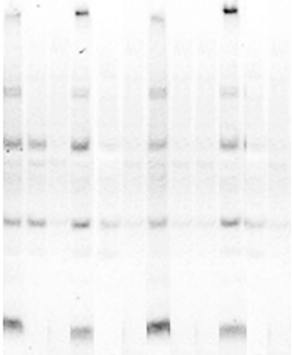

Fig. 3D

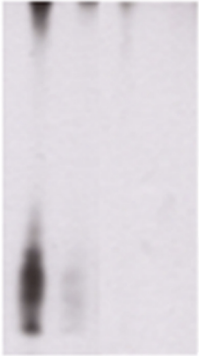

Fig. 3F

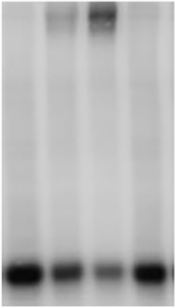

Fig. 2D

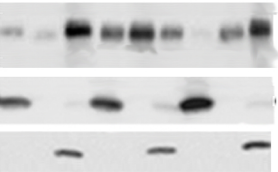

Fig. 3H

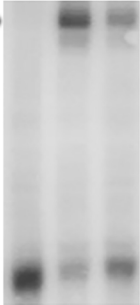

Fig. 6B

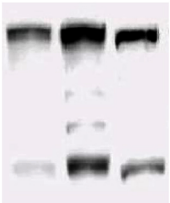

Fig. 6D

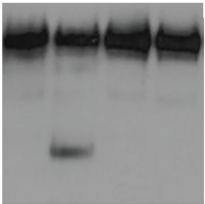

Supplement: Supplementary file 5 — supplementary data [file 41419_2022_4758_MOESM5_ESM.pdf]
